# Supplementary material for: Causal Relationship between Aging and Anorexia Nervosa: A White-Matter-Microstructure-Mediated Mendelian Randomization Analysis
Source: Biomedicines. 2024 Aug 16;12(8):1874. doi: 10.3390/biomedicines12081874 (PMC11351342; doi:10.3390/biomedicines12081874)
Supplement: Supplementary file 1 [file biomedicines-12-01874-s001.zip › Supplementary Figure/Figure S4/Singe SNP plot of MR analysis for TL to ubm-b-2055.pdf]

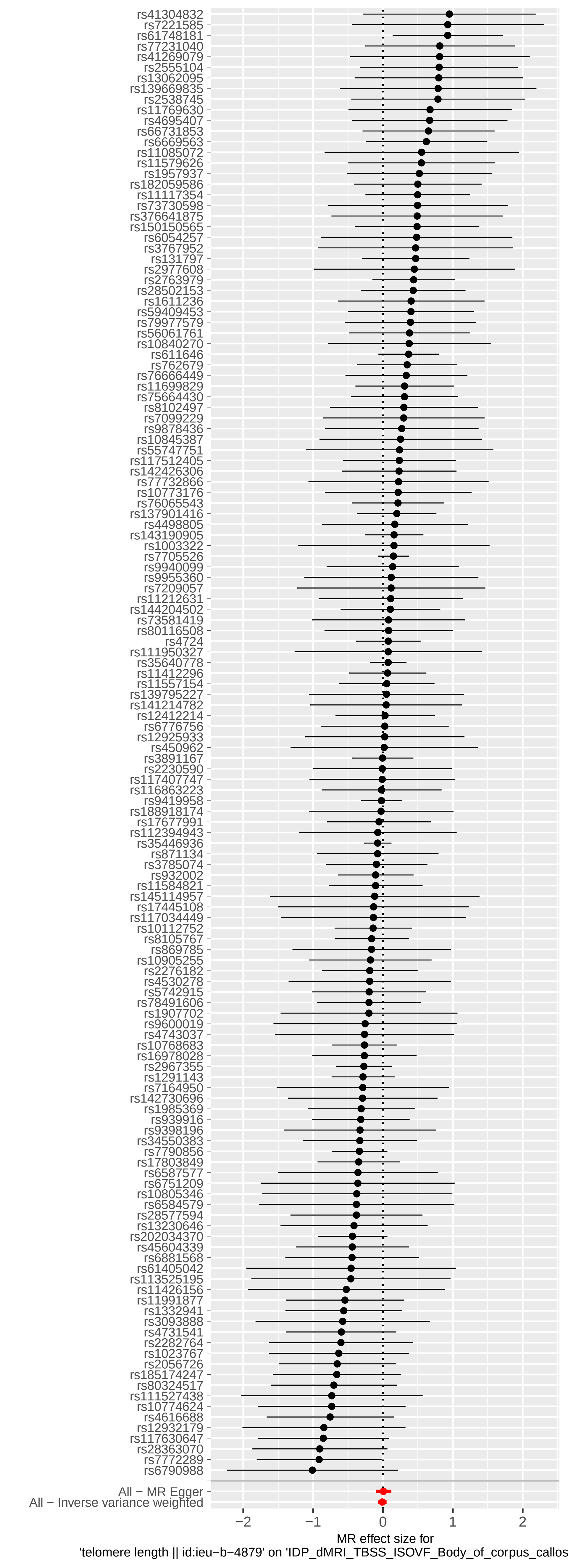
rs41304832
rs7221585
rs61748181
rs77231040
rs41269079
rs2555104
rs13062095
rs139669835
rs2538745
rs11769630
rs4695407
rs66731853
rs6669563
rs11085072
rs11579626
rs1957937
rs182059586
rs11117354
rs73730598
rs376641875
rs150150565
rs6054257
rs3767952
rs131797
rs2977608
rs2763979
rs28502153
rs1611236
rs59409453
rs79977579
rs56061761
rs10840270
rs611646
rs762679
rs76666449
rs11699829
rs75664430
rs8102497
rs7099229
rs9878436
rs10845387
rs55747751
rs117512405
rs142426306
rs77732866
rs10773176
rs76065543
rs137901416
rs4498805
rs143190905
rs1003322
rs7705526
rs9940099
rs9955360
rs7209057
rs11212631
rs144204502
rs73581419
rs80116508
rs4724
rs111950327
rs35640778
rs11412296
rs11557154
rs139795227
rs141214782
rs12412214
rs6776756
rs12925933
rs450962
rs3891167
rs2230590
rs117407747
rs116863223
rs9419958
rs188918174
rs17677991
rs112394943
rs35446936
rs871134
rs3785074
rs932002
rs11584821
rs145114957
rs17445108
rs117034449
rs10112752
rs8105767
rs869785
rs10905255
rs2276182
rs4530278
rs5742915
rs78491606
rs1907702
rs9600019
rs4743037
rs10768683
rs16978028
rs2967355
rs1291143
rs7164950
rs142730696
rs1985369
rs939916
rs9398196
rs34550383
rs7790856
rs17803849
rs6587577
rs6751209
rs10805346
rs6584579
rs28577594
rs13230646
rs202034370
rs45604339
rs6881568
rs61405042
rs113525195
rs11426156
rs11991877
rs1332941
rs3093888
rs4731541
rs2282764
rs1023767
rs2056726
rs185174247
rs80324517
rs111527438
rs10774624
rs4616688
rs12932179
rs117630647
rs28363070
rs7772289
rs6790988
All − MR Egger
All − Inverse variance weighted
−2
−1
0
1
2
MR effect size for
'telomere length || id:ieu−b−4879' on 'IDP_dMRI_TBSS_ISOVF_Body_of_corpus_callos
